# Relationship between patient-rated cleanliness and *Clostridioides difficile* standardized infection ratios in U.S. medicare-certified hospitals

**DOI:** 10.1017/ice.2025.10336

**Published:** 2026-03

**Authors:** Abigayle G. Rocca, William G. Greendyke, E. Yoko Furuya, Daniel E. Freedberg

**Affiliations:** 1 Department of Epidemiology, Columbia University Mailman School of Public Health, New York, NY, USA; 2 New York City Department of Health and Mental Hygiene, New York, NY, USA; 3 Department of Infectious Diseases, Columbia University Mailman School of Public Health, New York, NY, USA; 4 Division of Digestive and Liver Diseases, Columbia University Irving Medical Center, New York, NY, USA

## Abstract

**Objective:**

We evaluated whether patient perceptions of cleanliness are associated with objective measures of *Clostridioides difficile* infection (CDI), as an early indicator of facility-level CDI rates and prevention.

**Design:**

Cross-sectional analysis of Medicare-certified hospitals across the United States.

**Methods:**

Data from the CMS Hospital Compare website and U.S. Census Bureau from 2023 were analyzed using multivariate logistic regression models. The primary outcome was *C. difficile* standardized infection ratios (SIRs) compared to the national average. The primary exposure was patient-rated cleanliness star ratings from the Hospital Consumer Assessment of Healthcare Providers and Systems survey.

**Results:**

The population studied was 3,616 medicare-certified hospitals with an estimated 17,994,034 unique patient admissions. There was no association between better patient-rated cleanliness and improved CDI performance. Facilities with a 5-star cleanliness rating were not more likely to have an SIR less than or equal to the national average compared to those with a lower star rating. For every 1% increase in patients who reported their room and bathroom as always clean, the odds of CDI observed cases being higher than predicted increases by 4.2% (ie, increasing patient-related cleanliness was weakly associated with worse CDI performance).

**Conclusions:**

Patient-rated cleanliness was not associated with improved CDI performance in U.S. national hospital data. Findings were consistent across multiple operationalizations of cleanliness and CDI suggesting patient perceptions of cleanliness are not a strong indicator of CDI control measure performance.

## Introduction

*Clostridioides difficile* infection (CDI) is a leading cause of healthcare-associated diarrhea, with approximately 500,000 infections each year in the United States alone.^1,2^ The spread of *C. difficile* is influenced by factors at both the individual and facility levels. Individual risk factors include antibiotic exposure, advanced age, immunocompromised status, and length of stay in healthcare settings.^2,3^ Facility risk factors include antibiotic use and rate of *C. difficile* in the community (ie, importation rate).^4,5^ However, further understanding of facility-level predictors of *C. difficile* is essential for programmatic efforts to reduce CDI rates, which invariably must be deployed at the facility level.

At the facility level, environmental contamination and inadequate hand hygiene have been suggested as contributors to outbreaks of CDI,^5–7^ yet there is uncertainty regarding how to best operationalize factors such as these which are related to environment. The Centers for Medicare & Medicaid Services (CMS) monitors CDI rates in Medicare-certified hospitals, providing publicly accessible standardized infection ratios (SIRs) via their Hospital Compare website. CMS also collects patient experience data through the Hospital Consumer Assessment of Healthcare Providers and Systems (HCAHPS) survey, including patient perceptions of room cleanliness, summarized as star ratings. This patient-centered data related to hygiene therefore provides a potentially novel way of operationalizing environmental cleanliness which can be linked to standardized CDI rates across the nation. One prior study using the data from acute-care facilities in New York State found that higher patient-rated cleanliness was associated with lower rates of CDI,^8^ but there has been no national-level research exploring this relationship.

By analyzing a large national data set, this research aims to determine whether patient perceptions of cleanliness are associated with objective measures of CDI performance. Patient-rated cleanliness could inform understanding of *C. difficile* environmental transmission and potentially be used as a surrogate goal for interventions seeking to reduce CDI. We hypothesized that better patient-rated cleanliness would be associated with lower rates of CDI.

## Methods

### Data sources

The data utilized in this analysis were: (1) the Healthcare Associated Infections – Hospital and HCAHPS survey datasets from the 2023 CMS Hospital Compare Data; (2) the 2020 Decennial Census from the U.S. Census Bureau; and (3) the 2023 American Community Survey from the U.S. Census Bureau. All data are public and freely available. The Institutional Review Board of Columbia University Irving Medical Center waived approval of the study.

### Study population

The population studied was Medicare-certified hospitals across the U.S., from January 1 to December 31, 2023. The majority of hospitals in the United States are Medicare-certified, meaning they meet the minimum health and safety standards set by the federal government, evaluated by the State Survey Agency or a CMS-approved accreditation program. The facilities included were all contained within the HCAHPS survey dataset. From this data set, hospitals with missing values for *C. difficile* predicted cases or for patient-rated cleanliness were dropped.

### Outcomes

To enhance robustness, we examined three operationalizations of the CDI rate as binary variables. First, *C. difficile* standardized infection ratio (SIR) was compared to the 2023 national average SIR of 0.42,^9^ operationalized as less than or equal to, or greater than the national average *C. difficile* SIR. The model that predicts cases per facility, creating an SIR of 1 if predicted cases equal observed, was last updated in 2015^10^; our operationalization allows comparison of facilities and findings to be more relevant to current infection control practices. Second, we evaluated whether observed *C. difficile* cases were less than or equal to predicted cases. Third, *C. difficile* standardized infection ratio (SIR) was compared to the national benchmark as a binary variable, operationalized as meets or better than national benchmark performance. The outcome provides insight on facilities that exceeded infection control standards, based on the categorical variable provided by CMS Hospital Compare.

#### C. diff SIR compared to national average

The primary outcome assessed was the NHSN 2023 *C. difficile* SIR compared to the 2023 national average score of 0.42 as a binary variable. This was operationalized as “less than or equal to national average” versus “greater than national average.” SIR was calculated based on the observed and predicted *C. difficile* events at a given hospital from January 1, 2023 to December 31, 2023 Predicted *C. difficile* cases were calculated by the NHSN per facility based on national aggregate data. This was adjusted for each facility based off known risk factors for *C. difficile* that are out of a facilities’ control, including inpatient community-onset admission prevalence rate, CDI test type, number of ICU beds, facility type, total facility bed size, and other factors.^10^ SIRs are not available for facilities with fewer than1 predicted case of *C. difficile* for the year.

#### C. difficile observed cases compared to expected cases

A second measure of *C. difficile* infection rate was classified as the difference between expected and observed *C. difficile* cases. Facilities with a negative difference were classified as “observed *C. difficile* cases >predicted” and facilities with a positive difference or no difference were classified as “observed *C. difficile* cases ≤predicted.” This operationalization quantifies success based on the 2015 NHSN model and includes facilities without an SIR due to no expected cases.

#### C. diff SIR compared to national benchmark

The third outcome assessed was the NHSN 2023 C*. difficile* SIR compared to national benchmark as a binary variable. This was operationalized as “meets national benchmark” versus “better than national benchmark” designations per facility as provided in HCAHPS survey dataset.

### Exposures

The exposure of interest was patient-rated hospital room and bathroom cleanliness. This was captured through two operationalizations of the HCAHPS survey data. Cleanliness was analyzed (1) using a patient star rating and (2) as a continuous variable, based on the percent of patients that reported their room and bathroom as always clean.

#### Patient-rated cleanliness star rating

The primary exposure was patient-rated cleanliness star ratings collected via the HCAHPS survey. It was operationalized as a categorical ordinal variable, with values ranging from one to five with one being the least clean and five being the most clean. Star ratings were calculated using HCAHPS standardized methodology.^11^ Facilities were clustered into five groups based on the 5 possible star ratings.

#### Room and bathroom reported always clean, percentage

The percent of patients who reported their room and bathroom as always clean was analyzed as a continuous variable theoretically ranging from 0 to 100%. This value was provided for all facilities with submitted HCAHPS surveys and captures facilities excluded from receiving a star rating due to too few survey responses.

### Covariates

Covariates considered were the facility’s geographic region and demographic information for the patient population served, approximated through facility zip-code. The four standard CDC geographic regions included were the Northeast, Midwest, South, and West.^12^ Demographic covariates include race and ethnicity,^13^ median income,^14^ and population aged 65+.^15^ These were all categorized as binary variables with value per zipcode compared to the national average.

### Statistical analysis

Multivariate logistic regression models were used to analyze all *C. difficile* performance operationalizations. A multivariate logistic regression model was created for SIR compared to national average SIR of 0.42 as the outcome of interest with patient-rated cleanliness stars as the exposure of interest. A second multivariate logistic regression model was observed cases of *C. difficile* compared to expected with the primary exposure as the percent of patients who rated room as always clean. A third multivariate logistic regression model was created for SIR compared to national benchmark as the outcome of interest with patient-rated cleanliness stars as the exposure of interest. For all models, all covariates were included. All analysis was performed in StataNow/BE 18.5 for Mac (Intel 64-bit).

## Results

### Descriptive results

#### C. difficile standardized infection ratio

**Figure 1.**
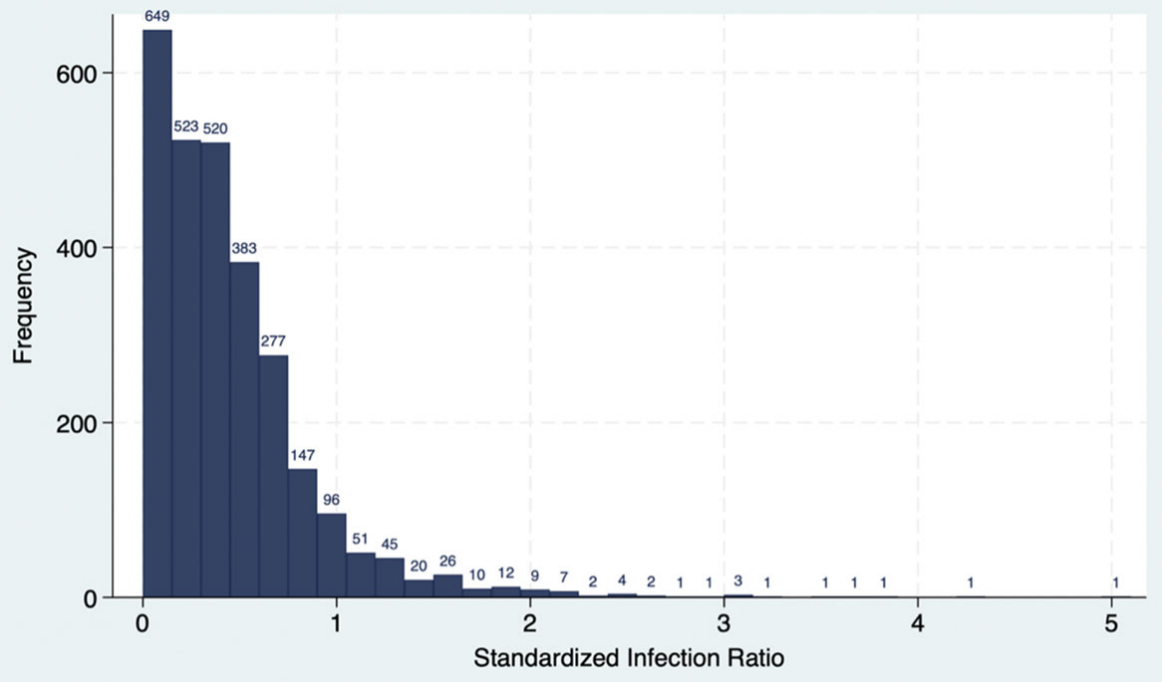
*C. difficile* standardized infection ratio histogram.

The total analyzed study population was 3,616 Medicare hospitals in the U.S. in 2023 who had an estimated, 17,994,034 unique patient admissions. We dropped 1,163 facilities from the full CMS Hospital Compare data set of 4,779 due to missing predicted cases or missing HCAHPS survey responses data. An additional 952 facilities were dropped due to missing SIR, missing cleanliness star rating, or insufficient number of HCAHPS survey responses to leave 2,664 facilities which were included in analysis of *C*. *difficile* SIR and cleanliness star rating. The *C*. *difficile* SIRs were right skewed with 58% of SIRs falling under the national average of 0.42 (Figure 1).

#### C. difficile standardized infection ratio and cleanliness star rating

**Figure 2.**
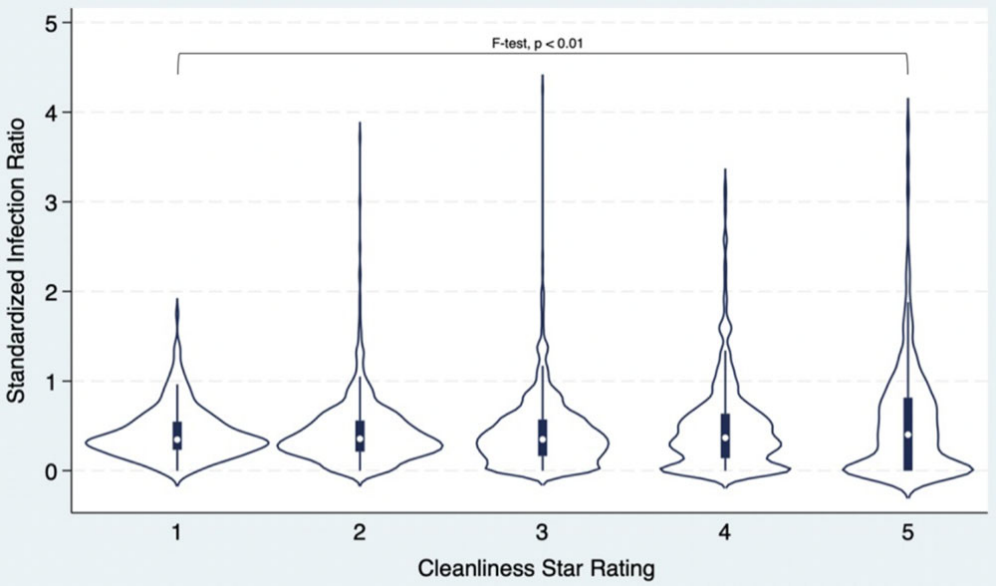
SIR by cleanliness star rating violin plot.

A violin plot was generated among the 2,664 facilities to illustrate the distribution of *C. difficile* SIRs across groups by cleanliness star ratings (Figure 2). The observed *C. difficile* SIRs were widely overlapping across these groups. Overall, 74% of facilities with a *C*. *difficile* SIR less than or equal to the national average of 0.42 received a 5-star cleanliness rating compared to 9.5% of facilities that had SIRs greater than the national average (*P* = .07, Table 1). In other words, the facilities that were performing better than the national average with respect to *C. difficile* rates were no different with respect to cleanliness ratings. However, only 24% of facilities with a *C*. *difficile* SIR better than the National Benchmark received a 5-star cleanliness rating compared to 17% of facilities that met (but did not exceed) the national benchmark (*P* < .01, Table 5, appendix). In other words, the facilities that were performing better with respect to *C. difficile* rates were slightly worse with respect to cleanliness ratings. Those with an SIR lower than the national average and better than the national benchmark were less likely to be in a zip-code containing older individuals and more likely to be in zip codes with high median income.

**Table 1.** Cleanliness star ratings and population characteristics by *C. difficile* SIR to national average

	*C. diff* SIR > national average *n* = 1,115 (42%)	*C. diff* SIR ≤ national average n = 1,549 (58%)	*P*-value
**Cleanliness star rating**			.07
1 Star – least clean (9.5%)	97 (8.7%)	156 (10%)	
2 Star (27%)	295 (26%)	431 (28%)	
3 Star (37%)	394 (35%)	579 (37%)	
4 Star (18%)	223 (20%)	269 (17%)	
5 Star – cleanest (8.3%)	106 (9.5%)	114 (7.4%)	
**Age**			< .01
Age 65+ years > national average	628 (56%)	774 (50%)	
Age 65+ years ≤ national average	487 (44%)	775 (50%)	
**Race**			
White alone > national average	516 (46%)	576 (37%)	< .01
Black alone > national average	264 (24%)	481 (31%)	< .01
Asian alone > national average	211 (19%)	287 (19%)	.796
Hispanic > national average	263 (24%)	420 (27%)	.040
Other > national average	558 (50%)	861 (56%)	.005
**Income**			.254
Median household income > national average	370 (33%)	547 (35%)	
Median household income ≤ national average	745 (67%)	1,002 (65%)	
**US geographic region**			< 0.01
Northeast	213 (19%)	214 (14%)	
Midwest	322 (29%)	342 (22%)	
South	304 (27%)	694 (45%)	
West	276 (25%)	299 (19%)	

Note. Covariates were operationalized as binary variables by comparing the percentage of the population within facilities’ ZIP codes to national average values: Age 65+ (17.3%),^15^ White (75.3%),^13^ Black (13.6%),^13^ Asian (6.4%),^13^ Other (4.7%),^13^ Hispanic (19.5%),^13^ and Median Household Income ($80,610).^14^ *P*-values were calculated using chi-square tests.

#### Observed cases and “Room Always Clean” sample

**Figure 3.**
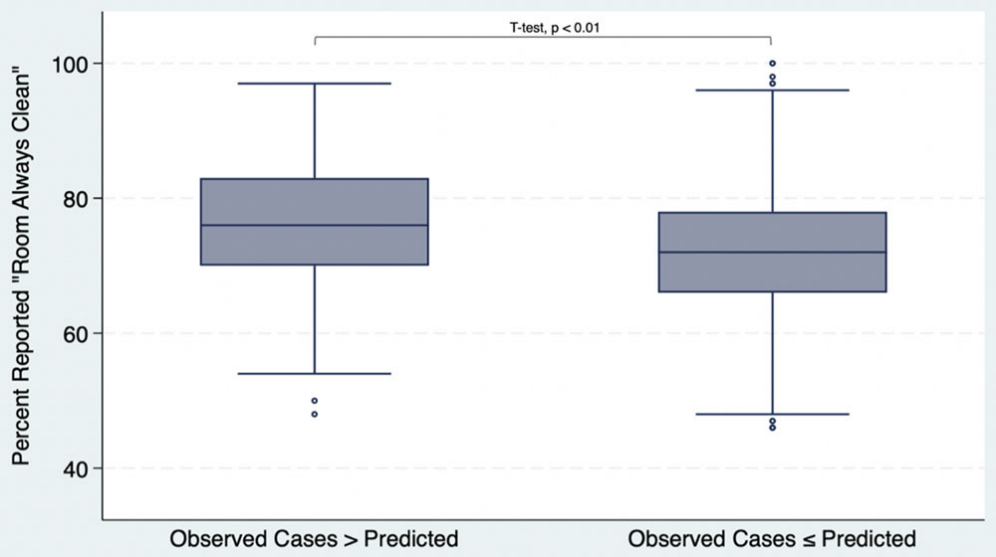
“Room Always Clean” and Observed vs Predicted cases box-and-whisker plot.

3,616 facilities were included in an analysis of observed cases compared to predicted cases and percent of patients who reported “room always clean.” This sample re-captured facilities that were excluded from the initial analysis due to having 0 predicted *C*. *difficile* cases (822 facilities), or too few HCAHPS survey responses (130 facilities). Facilities with more observed *C*. *difficile* than expected had a higher mean percentage of patients that reported their room and bathroom as always clean compared to facilities with observed cases that were less than or equal to the predicted amount (77% vs 72%, respectively, *P* < .01; Table 2 and Figure 3). Again, the directionality of this relationship was the reverse of what we had hypothesized. Facilities with greater than expected cases were more likely to be in a zip-code containing older individuals and less likely to be in zip codes with high median income.

**Table 2.** Patient reported cleanliness and population characteristics by observed *C. difficile* cases

	Observed C. diff cases >predicted *N* = 411 (11%)	Observed C. diff cases≤ predicted *N* = 3205 (89%)	*P*-value
**% of patients that reported room/bathroom:**	**Mean**	**Mean**	
Always clean	77%	72%	< .01*
Usually clean	16%	18%	< .01*
Sometimes or never clean	7.2%	9.5%	< .01*
**Age**			< .01
65+ years > national average	305 (74%)	1,789 (56%)	
65+ years ≤ national average	106 (26%)	1,416 (44%)	
**Race**			
% White alone > national average	265 (64%)	1,421 (44%)	< .01
% Black alone > national average	60 (15%)	878 (27%)	< .01
% Asian alone > national average	22 (5.4%)	518 (16%)	< .01
% Hispanic > national average	66 (16%)	767 (24%)	< .01
% Other > national average	140 (34%)	1,619 (50%)	< .01
**Income**			< .01
Median household income > national average	78 (19%)	967 (30%)	
Median household income ≤ national average	333 (81%)	2,238 (70%)	
**US geographic region**			< 0.01
Northeast	45 (11%)	435 (14%)	
Midwest	160 (39%)	903 (28%)	
South	111 (27%)	1,228 (38%)	
West	95 (23%)	639 (20%)	

Note. Covariates were operationalized as binary variables by comparing the percentage of the population within facilities’ ZIP codes to national average values: Age 65+ (17.3%),^15^ White (75.3%),^13^ Black (13.6%),^13^ Asian (6.4%),^13^ Other (4.7%),^13^ Hispanic (19.5%),^13^ and Median Household Income ($80,610).^14^ *P*-values were primarily calculated using chi-square tests;

*P-values calculated from t-tests.

### Logistic regression analysis

#### C. difficile SIR and cleanliness star rating

To examine the independent relationship between *C. difficile* SIR and cleanliness star rating, we built a logistic regression model. In the unadjusted model, there was a modest association between a rating of 5 stars (cleaner) and *C. difficile* SIR less than or equal to the national average (OR: 0.67, 95% CI: 0.46, 0.96; Table 3). Following the adjustment for all covariates, there was no association between cleanliness star rating and *C. difficile* SIR compared to the national average.

**Table 3.** Logistic regression model for risk factors to predict SIR **≤** national average

	Unadjusted		Adjusted	
**Cleanliness star rating**	**OR**	**95% CI**	**aOR**	**95% CI**
1 Star	*Referent*	*Referent*	*Referent*	*Referent*
2 Star	0.91	0.68–1.22	0.90	0.67–1.22
3 Star	0.91	0.69–1.21	0.94	0.70–1.25
4 Star	0.75	0.55–1.02	0.80	0.58–1.10
5 Star	0.67	0.46–0.96	0.78	0.53–1.13
**Age**				
65+ years > national average			0.86	0.72–1.03
**Race**				
White alone > national average			0.91	0.71–1.17
Black alone > national average			1.00	0.79–1.27
Asian alone > national average			0.94	0.74–1.20
Hispanic > national average			1.07	0.85–1.36
Other > national average			1.12	0.91–1.38
**Income**				
Median household income > national average			1.24	1.03–1.49
**Region**				
Northeast			*Referent*	*Referent*
Midwest			1.14	0.89–1.46
South			2.22	1.74–2.83
West			0.95	0.73–1.24

Note. Facilities with 1-star rating acting as referent. Adjusted models includes all listed covariates, operationalized as binary variables by comparing the percentage of the population within facilities’ ZIP codes to national average values: Age 65+ (17.3%),^15^ White (75.3%),^13^ Black (13.7%),^13^ Asian (6.4%),^13^ Other (4.7%),^13^ Hispanic (19.5%),^13^ and Median Household Income ($80,610).^14^

**Table 4.** Logistic regression model for risk factors to predict observed cases ≤ predicted

	Unadjusted		Adjusted	
**% of patients that reported room/bathroom:**	**OR**	**95% CI**	**aOR**	**95% CI**
Always clean	0.95	0.94 0.96	0.96	0.95–0.97
**Age**				
65+ years > national average			0.70	0.53–0.91
**Race**				
White alone > national average			1.15	0.80–1.65
Black alone > national average			1.51	1.02–2.25
Asian alone > national average			2.40	1.46–3.91
Hispanic > national average			1.06	0.71–1.58
Other > national average			1.74	1.29–2.33
**Income**				
Median household income > national average			1.47	1.11–1.96
**Region**				
Northeast			*Referent*	*Referent*
Midwest			0.84	0.58–1.21
South			1.14	0.77–1.69
West			0.50	0.34–0.76

Note. Percent of patients reporting room always clean is operationalized as a continuous variable, with odds ratios reflecting the change per 1% increase in reporting always clean. Adjusted models includes all listed covariates, operationalized as binary variables by comparing the percentage of the population within facilities’ ZIP codes to national average values: Age 65+ (17.3%),^15^ White (75.3%),^13^ Black (13.7%),^13^ Asian (6.4%),^13^ Other (4.7%),^13^ Hispanic (19.5%),^13^ and Median Household Income ($80,610).^14^

An additional logistic regression model was built to examine the relationship between *C. difficile* SIR by national benchmark category and cleanliness star rating. In this model, there was an association between ratings of 3–5 stars (cleaner) and worse *C. difficile* SIR (Table 6, appendix). As the patient-rated cleanliness star rating increased, the odds that a facility’s SIR was better than the national benchmark showed a stepwise decrease. Adjustment for age, race, income, and region had little impact on the odds ratio.

#### Room cleanliness and observed C. difficile cases

Similarly, a logistic regression model was built for patient-rated room cleanliness and observed *C. difficile* cases. In this model, room cleanliness was a continuous measure. For everyone 1% increase in patients who reported their room and bathroom as always clean, the odds of *C. difficile* observed cases being less than or equal to the facility’s predicted value decreased by 4% (aOR: 0.96, 95% CI: 0.95, 0.97). Adjusting for age, race, income, and region again had little impact on the odds ratio (crude OR: 0.95, aOR: 0.96).

## Discussion

We investigated the relationship between patient-rated cleanliness and *C. difficile* SIRs in Medicare-certified hospitals. We had hypothesized that better patient-rated cleanliness would be associated with lower rates of CDI. Contrary to this hypothesis, there was no association between better patient-rated cleanliness and improved *C. difficile* performance. Facilities with a 5-star cleanliness rating were not more likely to have a *C. difficile* SIR below (better than) the national average of 0.42 compared to those with lower ratings. Similarly, hospitals with higher percentages of patients reporting their room as always clean did not have increased odds of an observed *C. difficile* performance better than predicted. On the contrary, better patient-rated cleanliness was associated with marginally worse *C. difficile* performance when using national benchmark comparisons. These findings indicate that patient perceptions of cleanliness may not be a good surrogate for infection control measures targeting *C. difficile*.

Our results differ from Durant *et al*. (2021), which identified the expected inverse association between patient-perceived cleanliness and CDI rates within hospitals in New York State (ie, better patient-rated cleanliness was associated with better CDI rates). The difference in studies may be attributable to the broader national scope of our analysis and the different data used to quantify *C. difficile* infection control performance. Durant used the New York state Statewide Planning and Research Cooperative System (SPARCS) to calculate facility *C. difficile* infection rate. This data source had more detailed information about facilities compared to the Hospital Compare data set utilized in our analysis, which is pre-adjusted for facility-specific risk factors that are not publicly available. The associations seen in Durant were modest in strength and they may not generalize to the nation as a whole.

There are several potential explanations for why high patient-rated cleanliness scores did not predict better *C. difficile* performance in our analysis. First, patient-reported cleanliness ratings may not accurately reflect true cleanliness levels with respect to transmission of *C. difficile* spores. Patient-reported cleanliness is, by nature, subjective and may say more about a patient’s perception of room organization or overall appearance rather than true environmental cleanliness. For example, a recently renovated room might appear to be clean yet have a high burden of *C. difficile* spores. Second, patient surveys have a low completion rate, only 19.5% in our study population, and there may be respondent bias based on who chooses to complete the survey^16^; in other words, the completed surveys may not reflect the true patient perception of cleanliness within the population. Third, environmental burden of *C. difficile* spores may not be closely related to absence of dirt or staining. Spores are extremely hardy, and require substantial bleach exposure to kill. Rooms may be visually clean yet not have received sufficient bleach dwell time to effectively kill spores.^17,18^ Fourth, facilities with worse patient-reported cleanliness may have other facility-specific factors that serve as residual confounders and bias towards higher rates of CDI. For example, academic teaching institutions may be less focused on the patient experience and therefore perform worse on surveys, and also accept patients that are more medically complex and have a higher baseline risk for CDI.^19^ The national SIR for CDI, although standardized for patient factors such as age,^10^ may be unable to fully account for the patient-level differences between institutions. Last, *C. difficile* infection control measures go beyond solely maintaining effective environmental cleanliness protocols.^2,20^ Even if patient-rated cleanliness ratings are a reliable measure of true room cleanliness and sterilization, other factors in *C. difficile* infection control, such as antimicrobial stewardship practices, are not considered in this analysis and may be more important drivers of facilities’ *C. difficile* SIRs.

This study used secondary analyses conducted with different data operationalizations to increase the robustness of results. Utilizing the raw number values for observed cases, predicted cases, and percent of patients who reported room always clean, rather than the calculated SIR and star ratings from CMS hospital compare allowed for a larger sample size and let us interrogate the data in many ways. Throughout operationalizations, facilities that performed better than expected in terms of CDI rates had a lower mean percent of patients who reported their room as always clean compared to facilities that performed worse than expected in terms of CDI rates (ie, the opposite of our initial hypothesis). Multiple analyses reinforced the primary finding that facilities with higher cleanliness ratings do not necessarily have better *C. difficile* infection control performance.

This study has several strengths and also limitations. The analysis was conducted on a large national sample of medicare-certified hospitals and used multiple operationalizations of data to enhance robustness of findings. However, the data did not capture hospital-specific infection control measures, had already been previously adjusted by NHSN, and was grouped by hospital systems. Response bias in the HCAHPS survey and potential unmeasured confounders, such as differences in facility antimicrobial stewardship practices or in patient factors, may also influence results.

In sum, this study using national U.S. data from 2023 found no association between higher patient-rated cleanliness and better *C. difficile* performance, contradicting the one prior study to examine this novel measure for hospital hygiene. These results emphasize the difficulty in accurately measuring hygiene with respect to transmission of *C. difficile* and underscore the complexity of healthcare-associated CDI, an important problem which cannot be simplified as solely due to “dirty rooms.”

## Supporting information

Rocca et al. supplementary material Rocca et al. supplementary material

## Supplementary material

The supplementary material for this article can be found at https://doi.org/10.1017/ice.2025.10336.
