# Supplementary material for: Relationship between patient-rated cleanliness and Clostridioides difficile standardized infection ratios in U.S. medicare-certified hospitals
Source: Infect Control Hosp Epidemiol. 2025 Dec 12;47(3):257–64. doi: 10.1017/ice.2025.10336 (PMC12932921; doi:10.1017/ice.2025.10336)
Supplement: Rocca et al. supplementary material [file S0899823X2510336Xsup001.docx]

**Table 1:** Cleanliness star ratings and population characteristics by *C. difficile* SIR national benchmark

|  | ***C. diff* SIR Meets National Benchmark**  **n = 1,062 (40%)** | ***C. diff* SIR Better than National Benchmark**  **n = 1,589 (60%)** | **p-value** |
| --- | --- | --- | --- |
| **Cleanliness Star Rating** |  |  | <0.01 |
| 1 Star – least clean (9.5%) | 62 (5.8%) | 190 (12%) |  |
| 2 Star (27%) | 192 (18%) | 530 (33%) |  |
| 3 Star (37%) | 354 (33%) | 615 (39%) |  |
| 4 Star (18%) | 274 (26%) | 216 (14%) |  |
| 5 Star – cleanest (8.3%) | 180 (17%) | 38 (2.4%) |  |
| **Age** |  |  | <0.01 |
| Age 65+ years > National average | 692 (65%) | 704 (44%) |  |
| Age 65+ years ≤ National average | 370 (35%) | 885 (56%) |  |
| **Race** |  |  |  |
| White alone > National average | 612 (58%) | 475 (30%) | <0.01 |
| Black alone > National average | 196 (18%) | 545 (34%) | <0.01 |
| Asian alone > National average | 113 (11%) | 383 (24%) | <0.01 |
| Hispanic > National average | 215 (20%) | 463 (29%) | <0.01 |
| Other > National average | 473 (45%) | 939 (59%) | <0.01 |
| **Income** |  |  | <0.01 |
| Median Household Income > National average | 290 (27%) | 624 (39%) |  |
| Median Household Income ≤ National average | 772 (73%) | 965 (61%) |  |
| **US Geographic Region** |  |  | <0.01 |
| Northeast | 168 (16%) | 258 (16%) |  |
| Midwest | 344 (32%) | 318 (20%) |  |
| South | 320 (30%) | 670 (42%) |  |
| West | 228 (21%) | 341 (21%) |  |
| The exposure in this table was cleanliness star rating and the outcome was binary outcome of SIR meets vs. better than national benchmark. .Covariates were operationalized as binary variables by comparing the percentage of the population within facilities’ ZIP codes to national average values: Age 65+ (17.3%),^18^ White (75.3%),^19^ Black (13.6%),^19^ Asian (6.4%),^19^ Other (4.7%),^19^ Hispanic (19.5%),^19^ and Median Household Income ($80,610).^20^ P-values were calculated using chi-square tests. | | | |

**Table 2:** Logistic regression model for risk factors to predict SIR better than national benchmark

|  | **Unadjusted** | | **Adjusted** | |
| --- | --- | --- | --- | --- |
| **Cleanliness Star Rating** | **OR** | **95% CI** | **aOR** | **95% CI** |
| 1 Star | *Referent* | *Referent* | *Referent* | *Referent* |
| 2 Star | 0.91 | 0.65 1.25 | 0.88 | 0.63 1.24 |
| 3 Star | 0.57 | 0.41 0.78 | 0.55 | 0.40 0.77 |
| 4 Star | 0.26 | 0.18 0.36 | 0.27 | 0.19 0.39 |
| 5 Star | 0.07 | 0.04 0.10 | 0.09 | 0.06 0.14 |
| **Age** |  |  |  |  |
| 65+ years > National average |  |  | 0.63 | 0.52 0.77 |
| **Race** |  |  |  |  |
| White alone > National average |  |  | 0.60 | 0.52 0.76 |
| Black alone > National average |  |  | 1.15 | 0.89 1.50 |
| Asian alone > National average |  |  | 1.58 | 1.20 2.08 |
| Hispanic > National average |  |  | 0.94 | 0.72 1.22 |
| Other > National average |  |  | 1.14 | 0.91 1.43 |
| **Income** |  |  |  |  |
| Median Household Income > National average |  |  | 1.73 | 1.42 2.11 |
| **Region** |  |  |  |  |
| Northeast |  |  | *Referent* | *Referent* |
| Midwest |  |  | 0.77 | 0.58 1.02 |
| South |  |  | 1.26 | 0.96 1.64 |
| West |  |  | 0.68 | 0.51 0.92 |
| The exposure in this model was cleanliness star rating and the outcome was binary outcome of SIR meets vs. better than national benchmark. Facilities with 1-star rating acting as referent. Adjusted models includes all listed covariates, operationalized as binary variables by comparing the percentage of the population within facilities’ ZIP codes to national average values: Age 65+ (17.3%),^18^ White (75.3%),^19^ Black (13.7%),^19^ Asian (6.4%),^19^ Other (4.7%),^19^ Hispanic (19.5%),^19^ and Median Household Income ($80,610).^20^ | | | | |
